# Efficacy of a novel percutaneous pedicle screw fixation and vertebral reconstruction versus the traditional open pedicle screw fixation in the treatment of single-level thoracolumbar fracture without neurologic deficit

**DOI:** 10.3389/fsurg.2022.1039054

**Published:** 2023-01-06

**Authors:** Lining Rui, Fudong Li, Cao Chen, Yuan E, Yuchen Wang, Yanhong Yuan, Yunfeng Li, Jian Lu, Shengchang Huang

**Affiliations:** ^1^ Department of Spinal Surgery, Wujin Hospital of Traditional Chinese Medicine, Changzhou, China; ^2^ Department of Orthopaedic Surgery, Spine Center, Shanghai Changzheng Hospital, Naval Medical University, Shanghai, China; ^3^ Department of Clinical Medicine, Nanjing Medical University, Nanjing, China; ^4^ Department of Sports Medicine, Wujin Hospital of Traditional Chinese Medicine, Changzhou, China

**Keywords:** thoracolumbar fracture, percutaneous pedicle screw fixation and vertebral reconstruction, anterior vertebral height, open pedicle screw fixation, vertebral reconstruction

## Abstract

**Objective:**

The aim of this study was to compare the efficacy and safety of a novel percutaneous pedicle screw fixation and vertebral reconstruction (PPSR) vs. that of open pedicle screw fixation (OPSF) in the treatment of thoracolumbar fractures.

**Methods:**

This retrospective study enrolled 153 patients who underwent PPSR and 176 patients who received OPSF. Periprocedural characteristics, radiographic parameters, and clinical outcomes were compared between the two groups.

**Results:**

The operation duration was 93.843 ± 20.611 in PPSR group and 109.432 ± 11.903 in OPSF group; blood loss was 131.118 ± 23.673 in PPSR group and 442.163 ± 149.701 in OPSF group, incision length was 7.280 ± 1.289 in PPSR group and 14.527 ± 2.893 in OPSF group, postoperative stay was 8.732 ± 1.864 in PPSR group and 15.102 ± 2.117 in OPSF group, and total hospitalization costs were 59027.196 ± 8687.447 in PPSR group and 73144.432 ± 11747.567 in OPSF group. These results indicated that these parameters were significantly lower in PPSR compared with those in OPSF group. No significant difference was observed in the incidence of complications between the two groups. The radiographic parameters including height of the anterior vertebra, Cobb angle, and vertebral wedge angle were better in PPSR group than in OPSF group. Recovery rate of AVH was 0.449 ± 0.079 in PPSR group and 0.279 ± 0.088 in OPSF group. Analysis of clinical results revealed that during postoperative period, the VAS and ODI scores in PPSR group were lower than those in OPSF group.

**Conclusions:**

Collectively, these results indicated that PPSR more effectively restored the height of anterior vertebra and alleviated local kyphosis compared with OPSF. Moreover, the VAS and ODI scores in PPSR group were better than those of OPSF group.

## Introduction

Spine injuries, especially thoracolumbar fractures caused by various factors such as accidents are on the rise. Thoracolumbar fractures account for more than 50% of all spinal fractures (1), and pose a huge economic burden to the society and families. Patients with stable spine fractures can be treated conservatively, while surgery is needed for severe damage of the vertebral column, kyphotic deformity, or neurological disorders (2). At present, operative indications of type A thoracolumbar fracture without neurologic deficit include: (1) kyphotic deformity > 15–20° (compared with normal angle); (2) the loss of vertebral body height > 50%. The objective of surgery for type A thoracolumbar fracture is to restore the vertebral body height, correct the Cobb angle, and correct the kyphotic deformity. Although the open pedicle screw fixation (OPSF) system is one of the most effective methods for treating thoracolumbar fractures, it suffers from several limitations. These include the difficulty in accurate placement of the screws and the necessity to obtain a wide exposure of the facets and transverse processes through dissection of the paravertebral muscles, which play a pivotal role in maintaining spinal stability. The traditional OPSF often causes excessive blood loss, requires prolonged hospital stays, and is expensive (3). In addition, vertebral fracture reduction under the conventional OPSF is not satisfactory because the anterior vertebral height (AVH) is restored and the Cobb angle corrected through longitude traction of the titanium rod. These disadvantages have limited the widespread use of OPSF in treating thoracolumbar fractures.

Magerl developed and reported for the first time in 1982 a minimally invasive percutaneous pedicle screw technique combined with external fixation (4). This technique minimized surgical trauma and decreased the surgical duration of spine surgery. In comparison to OPSF, the percutaneous pedicle screw technique has the advantages of less bleeding, shorter operative time, and lower visual analog scale (VAS) score after surgery (5). However, neither OPSF nor the traditional percutaneous pedicle screw fixation can restore the normal levels of postoperative AVH and the Cobb angle. It has been reported that the postoperative AVH and the correction of Cobb angle are gradually lost with time, in patients who receive the OPSF or the traditional percutaneous pedicle screw fixation (5–7) due to failure of reconstruction of the anterior column. According to the three-column spinal theory, a stable anterior spinal column is essential for normal spinal biomechanics (8). Although the traditional pedicle screw fixation technique can restore the stability of the posterior columns of the injured vertebra, it fails to restore the anterior spinal column. Consequently, the resulting biomechanical instability is often associated with low back pain and requires revision surgery. Therefore, it is crucial to develop a novel percutaneous pedicle screw fixation technique that is capable of reconstructing the anterior column.

Based on traditional percutaneous pedicle screw fixation, we devised a new technique named percutaneous pedicle screw fixation and vertebral reconstruction (PPSR). This technique can be used in the reconstruction of the anterior column by distracting the involved vertebra and providing bone grafting to promote vertebral healing. PPSR was used for treating patients who had vertebral compression fracture. This retrospective study was carried out to compare the efficacy of PPSR and the traditional OPSF. In this study, medical records of patients with thoracolumbar fractures were retrospectively reviewed. Clinical features and surgical outcomes of the two types of surgeries were compared. The results indicated that OPSF and PPSR give both short-term and long-term benefits. Other advantages include shorter operating time, reduced financial burden, and preservation of anterior vertebral height and the local vertebral Cobb angle. Therefore, the novel PPSR is a reliable method for treating thoracolumbar fractures and offers many benefits to patients.

## Materials and methods

### Patients

This was a retrospective cohort study that was approved by the Ethics Committee of the Wujin Hospital of Traditional Chinese Medicine (KY-S-2019002). This study has been revised according to the STROBE checklist which is provided in the supplemental file (Supplementary File 1). A total of 656 patients with thoracolumbar fracture treated from July 2018 to June 2022 were enrolled. Inclusion criteria were as follows: (1) thoracolumbar fractures (T10–L2) caused by trauma, confirmed by imaging; (2) single-segment vertebral fracture; (3) AO type A fracture; (4) the fracture occurred within one week prior to surgery; (5) no prior history of spinal fracture; (6) no spinal canal occupation; (7) had under gone PPSR or OPSF; (8) with complete case records. Exclusion criteria: (1) severe osteoporosis; (2) neurological deficit; (3) ankylosing spondylitis or spine malformation; (4) primary or secondary tumor of the spine. Thoracolumbar fracture was diagnosed using x-ray, computed tomography (CT) and magnetic resonance imaging (MRI). All surgical procedures were performed by the same senior spine surgeon. Data related to clinical follow-up were collected through either outpatient follow-ups or telephone contacts. Specifically, most type A1 patients who strongly demanded PPSR or OPSF were included in this study. Type A2 or A3 patients with vertebral compression fracture who accepted PPSR or OPSF were also included in this study. Eventually, 329 patients were enrolled in this study. Vertebral reconstruction in 153 patients was carried out using PPSR, while 176 patients underwent OPSF. All the patients were informed of the surgical treatment procedures as well as the benefits and risks of the surgeries. Upon admission, all the patients consented to the use of their data for scientific research. The procedures were performed by the same surgical team.

### Operative procedures

**Figure 1:**
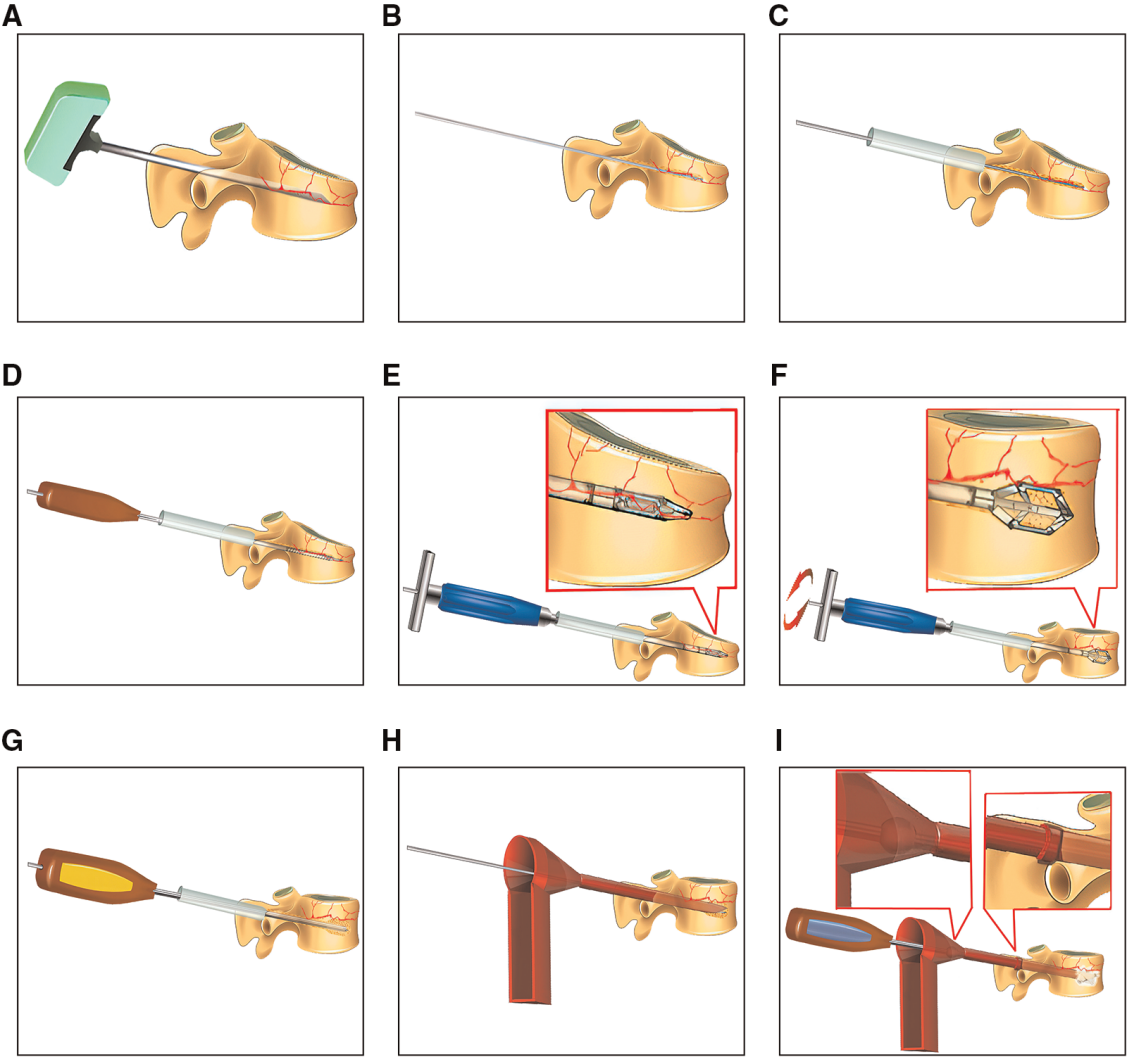
Illustration of PPSR procedures. (**A**) A puncture needle inserted into the appropriate depth of the fractured vertebra as examined by fluoroscopy. (**B**) A guide needle inserted into the vertebral body. (**C**) A hollow sleeve created to protect the surrounding soft tissues and guide the devices. (**D**) A rotary chisel applied to establish a channel for the percutaneous distractor. (**E,F**) The percutaneous vertebral distractor inserted into the fractured vertebra; the vertebral distraction was achieved by turning the percutaneous vertebral distractor. (**G**) A channel dilator was used to enlarge the channel for bone grafting. (**H**) Bone grafting through a funnel. (**I**) A bone grafting rod was used to push the bone graft materials into the injured vertebra. An accessory ball was applied to limit the depth range of the bone grafting rod. Range of the bone grafting rod.

**Figure 2:**
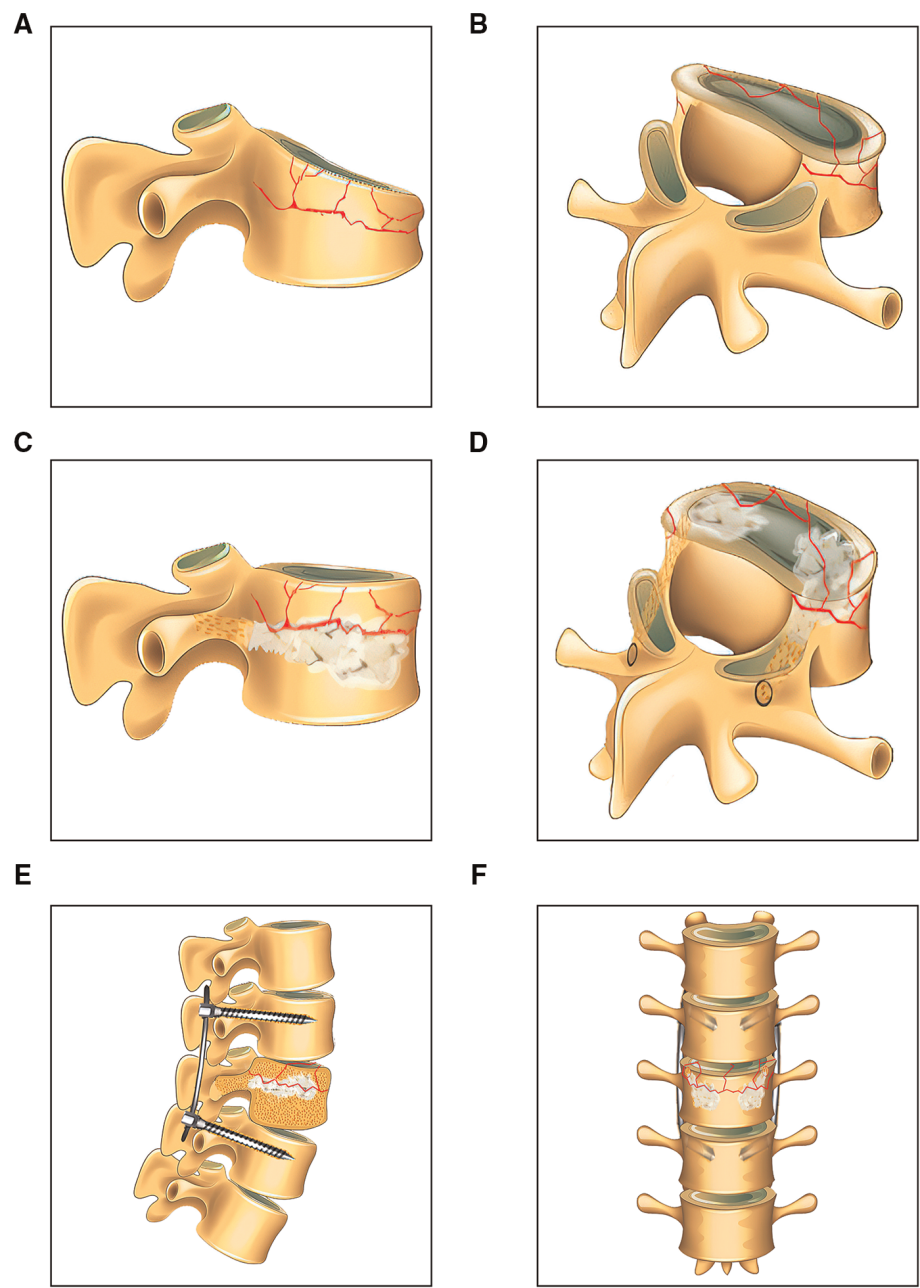
The simulation diagram of treatment outcomes in patients who underwent PPSR. (**A,B**) The preoperative sagittal and oblique diagram of the fractured vertebra. (**C,D**) The postoperative sagittal and oblique diagram of the vertebra. (**E,F**) The images of postoperative spinal segments in coronal and sagittal planes.

**Figure 3:**
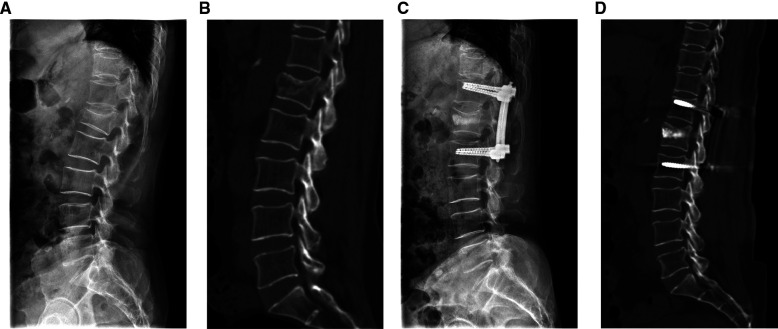
A patient treated with PPSR. (**A,B**) The preoperative sagittal radiograph (**A**) and CT image (**B**). (**C,D**) The postoperative sagittal radiograph (**C**) and CT image (**D**).

The procedures for PPSR are presented in Figure 1. The PPSR procedure was as follows. (1) Preoperative positioning was first performed. Patients were placed in prone position with their shoulder and pelvis slightly raised after sedation with general anesthesia. Appropriate incisions for inserting percutaneous pedicle screw placement was confirmed by C-arm fluoroscopy. The pedicle positions of the fractured vertebra and the adjacent vertebrae were marked on the body surface. (2) Routine disinfection was carried out and sterile drapes were applied. Four longitudinal skin incisions (1.5–2 cm) were made at 1 cm lateral to the projection area of the adjacent vertebrae pedicles. Guide pins were inserted into the vertebra through the pedicles using C-arm fluoroscopy. Pedicle screws were implanted and their positions confirmed with fluoroscopy. The pre-bending rods were connected to pedicle screws, but not completely fixed. (3) Two longitudinal incisions (1.5–2 cm) were performed at 2 cm lateral to the projection area of the fractured vertebra pedicles. The novel distractor developed by our group was inserted into the fractured vertebra. The anterior end was close to the position of the superior endplate collapse. The collapsed endplates were distracted by turning the distractor. The same maneuver was carried out on the opposite side (4). Allogenous bone was used for bone grafting. A C-arm fluoroscopy revealed that the vertebral body height of the collapsed vertebra recovered and the rods were completely fixed with screws. The simulation diagram of the treatment outcome in patients underwent PPSR was presented (Figure 2). PPSR was used on a female patient with an L1 vertebral compression fracture and the preoperative and postoperative radiographic images are presented in Figure 3. The AVH increased from 52.3% preoperatively to 98.8% postoperatively. The VWA decreased from 13.4° preoperative to 4.2° postoperative and the Cobb angle was corrected from 24.5° preoperatively to 6.3° postoperatively. No symptoms of discomfort were reported by the time of last follow-up. OPSF procedures are based on previously published literature (9).

### Clinical parameters

Data collected from the medical records included age, gender, Body Mass Index (BMI), hypertension, diabetes, operative duration, blood loss, total incision length, postoperative stay, total hospitalization costs, fracture segment, the back and leg VAS score (0–10) and the Oswestry Disability Index (ODI) (0%–100%). VAS and ODI were collected at the time of admission, and at 1 week, 3 months, and 12 months after surgery through subsequent visit or telephone follow-up. All the clinical parameters were assessed by two junior attending physicians, and if their findings differed, a second opinion was sought from a senior physician.

### Radiographic parameters

**Figure 4:**
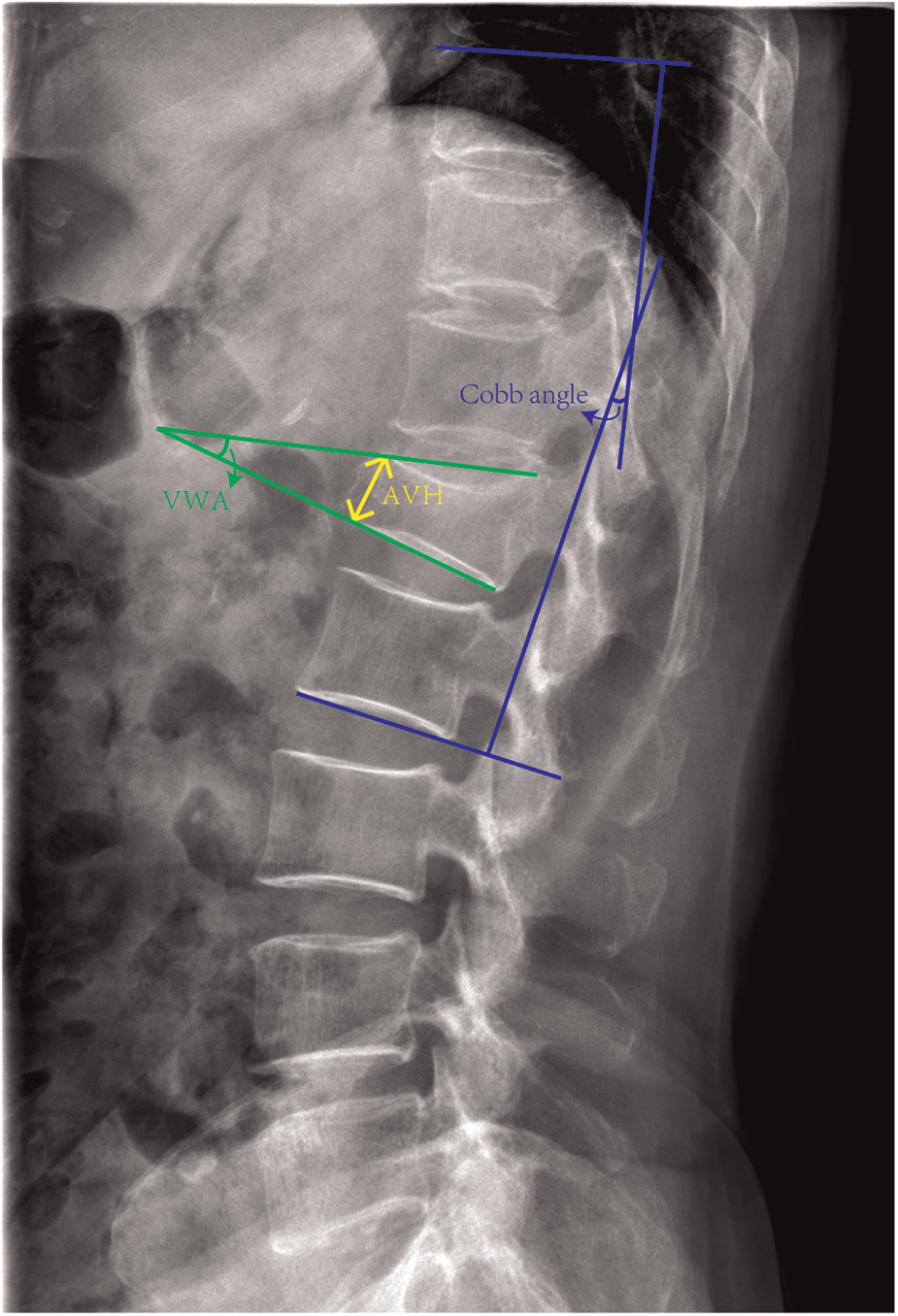
The definition of AVH, VWA and Cobb angle. The AVH refers to the height of the anterior vertebra. The VWA was defined as the angle between superior and inferior endplates of the fractured vertebra. The Cobb angle was considered as the angle between the upper endplate of T10 and the lower endplate of L2.

To evaluate the restoration of vertebral height and spinal curvature, the recovery rates of AVH, vertebral wedge angle (VWA), and Cobb angle were measured. The imaging findings were interpreted by two spine surgeons who had more than 10 years of clinical practice. If results from the two surgeons were inconsistent, the imaging findings would be evaluated by the professor with higher seniority in our team. Before surgery and 12 months after operation, all patients were subjected to radiographs, CT scans, and MRIs of the spine and the AVH, VWA, and Cobb angle were assessed. Recovery rates of AVH (%) = (postoperative AVH of the injured vertebra—pre-operative AVH of the injured vertebra)/[(AVH of the superior vertebra + AVH of the inferior vertebra)/2] (Figure 4). VWA was defined as the angle between superior and inferior endplates of the fractured vertebra (Figure 4). The Cobb angle was regarded as the angle between the upper endplate of T10 and the lower endplate of L2 on a lateral x-ray (Figure 4).

### Statistical analysis

All the data were measured by at least two surgeons. Data in this study were analyzed by using SPSS (version 25.0) and Graphpad prism (version 8). Continuous variables were expressed as mean ± SD, and the enumeration data were was expressed as percentage. Independent sample t test was used for group comparisons for data, including BMI, operative duration, blood loss, incision length, total hospitalization costs, recovery rates of AVH, local Cobb angle, and VWA. Categorical variables were investigated by the *χ*^2^ test. Normality was checked using the Shapiro–Wilk normality test. A two-sample *t*-test was used for normally distributed data, while Mann-Whitney *U* test was used for non-normally distributed data. A *P* < 0.05 was considered statistically significant.

## Results

### Demographic and baseline characteristics

The demographic and baseline characteristics of the two groups were shown in Table 1. A total of 329 patients were included in this study. PPSR group had 153 patients (69 females and 84 males) and while the OPSF group 176 patients (81 females and 95 males). Average age of patients in the PPSR group at the time of surgery was 51.020 ± 11.540 and 52.181 ± 13.842 years in the OPSF group. There was no significant difference in baseline and demographic characteristics including gender, age, BMI, hypertension, diabetes, and fracture segment between the two groups (Table 1). T12 (24.836% in PPSR group and 26.136% in OPSF group) was the most frequently involved vertebra, followed by L1 (22.876% in in PPSR group and 22.159% in OPSF group).

**Table 1:** Demographic and baseline characteristics of the patients.

Variables	PPSR (*n* = 153)	OPSF (*n* = 176)	*T* value	*P* value
Gender (Female) *n*, %	69 (45.098)	81 (46.023)	0.028	0.867
Age, year	51.020 ± 11.540	52.181 ± 13.842	−0.82	0.413
BMI	25.844 ± 3.054	25.607 ± 3.776	0.622	0.535
Hypertension, *n*, %	25 (16.340)	28 (15.909)	0.011	0.916
Diabetes, *n*, %	18 (11.765)	22 (12.500)	0.041	0.839
**Fracture segment, *n*, %**				
T10	20 (13.072)	25 (14.205)	0.089	0.766
T11	28 (18.301)	33 (18.750)	0.011	0.917
T12	38 (24.836)	46 (26.136)	0.073	0.787
L1	35 (22.876)	39 (22.159)	0.024	0.877
L2	32 (20.915)	33 (18.750)	0.242	0.623
**Types of fracture, *n*, %**				
A1	83 (54.248)	98 (55.682)	0.068	0.794
A2	32 (20.915)	35 (20.231)	0.053	0.817
A3	38 (24.837)	43 (24.432)	0.007	0.932

### Periprocedural characteristics of the patients in the PPSR group and the OPSF group

Perioperative data are shown in Table 2. Significant differences in features such as operation duration, blood loss, incision length, postoperative stay, and the total hospitalization costs were observed between the PPSR and the OPSF groups. Operative duration was 93.843 ± 20.611 min in PPSR group vs. 109.432 ± 11.903 min in OPSF group (*P* < 0.001). Volume of intraoperative blood loss was 131.118 ± 23.673 ml in PPSR group and 442.163 ± 149.701 ml in OPSF group (*P* < 0.001). The total length of skin incision was 7.280 ± 1.289 cm and 14.527 ± 2.893 cm in the PPSR group and the OPSF group (*P* < 0.001), respectively. The postoperative hospital stay of the patients was 8.732 ± 1.864 days in the PPSR and 15.102 ± 2.117 days in the OPSF group (*P* < 0.001). The cost of hospitalization in the PPSR group (59027.196 ± 8687.447 yuan) was significantly lower than that in the OPSF group (73144.432 ± 11747.567 yuan). Complications that were observed between the two groups did not differ considerably. Furthermore, no patients with single-level thoracolumbar fracture who received PPSR or OPSF experienced pulmonary embolism or bone material leakage after surgery.

**Table 2:** Periprocedural data for PPSR and OPSF groups.

Variables	PPSR (*n* = 153)	OPSF (*n* = 176)	*T* value	*P* value
**Operative duration, min**	93.843 ± 20.611	109.432 ± 11.903	−8.531	<0.001
**Blood loss, ml**	131.118 ± 23.673	442.163 ± 149.701	−25.421	<0.001
**Incision length, cm**	7.280 ± 1.289	14.527 ± 2.893	−28.610	<0.001
**Postoperative stay, day**	8.732 ± 1.864	15.102 ± 2.117	−28.770	<0.001
**Total hospitalization costs, yuan**	59027.196 ± 8687.447	73144.432 ± 11747.567	−12.237	<0.001
**Complications**				
Postoperative hematoma, *n*, %	1 (0.654)	2 (1.136)	0.211	0.646
Infection, *n*, %	1 (0.654)	3 (1.705)	0.753	0.386
Pedicle breach, *n*, %	2 (1.307)	1 (0.568)	0.495	0.482
Loose nut, *n*, %	2 (1.307)	1 (0.568)	0.495	0.482

### Preoperative and postoperative radiographic results

The radiographic results of the PPSR group and the OPSF group were presented in the Table 3. The AVH was used to estimate the severity of vertebra fracture and the recovery of the vertebral structure. The preoperative AVH was 1.477 ± 0.238 cm in the PPSR group and 1.440 ± 0.167 cm in the OPSF group, indicating that the pre-operative AVH between the two groups did not differ significantly (*P* = 0.100). The results of the AVH at 12-month after surgery demonstrated that the AVH in the PPSR group (2.713 ± 0.176 cm) was significantly higher than that in the OPSF group (2.231 ± 0.166 cm, *P* < 0.001). In addition, the recovery rate of AVH in patients that underwent PPSR (0.449 ± 0.079) was notably better than in patients who underwent OPSF (0.279 ± 0.088, *P* < 0.001). In addition, the degree of spinal kyphosis was evaluated by local Cobb angle. The preoperative Cobb angles between the two groups were statistically consistent (*P* > 0.05). The local Cobb angle at the 12-month after surgery between the PPSR group (7.570 ± 1.422°) and the OPSF group (12.631 ± 1.421°) was statistically significant (*P* < 0.001). Moreover, the VWA was also measured to further assess the efficacy of the surgeries used for the vertebral reconstruction. The preoperative VWAs of the two groups did not differ. The results showed that the VWA in the PPSR group (6.747 ± 1.323**°**) was greater than that in the OPSF group (9.938 ± 1.385°, *P* < 0.001). This finding demonstrated that PPSR are more effective than conventional OPSF in reconstructing fractured vertebra.

**Table 3:** Preoperative and postoperative AVH, cobb angle, and VWA in PPSR and OPSF groups.

Variables	PPSR (*n* = 153)	OPSF (*N* = 176)	*T* value	*P* value
**AVH**				
Pre-operative, cm	1.477 ± 0.238	1.440 ± 0.167	1.648	0.100
12-months after surgery, cm	2.713 ± 0.176	2.231 ± 0.166	25.511	<0.001
Recovery rates of AVH	0.449 ± 0.079	0.279 ± 0.088	18.314	<0.001
**Cobb angle, °**				
Pre-operative	24.137 ± 0.573	24.128 ± 0.594	0.147	0.883
12-month after surgery	7.570 ± 1.422	12.631 ± 1.421	−32.191	<0.001
**VWA, °**				
Pre-operative	12.250 ± 2.562	12.568 ± 1.663	−1.349	0.178
12-months after surgery	6.747 ± 1.323	9.938 ± 1.385	−21.278	<0.001

### The clinical outcomes between the PPSR group and the OPSF group

**Figure 5:**
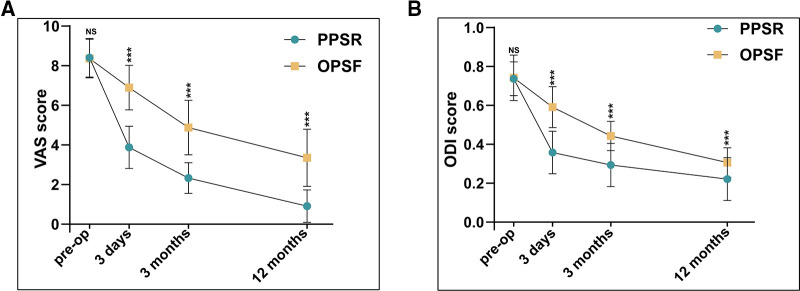
Comparison of clinical outcomes between the PPSR group and OPSF group. (**A**) The VAS scores between the PPSR group and OPSF group. (**B**) The ODI scores between the PPSR group and OPSF group. NS indicates no significance. Data are presented as the mean ± SD. *: *P* < 0.05, **: *P* < 0.01, ***: *P* < 0.001.

The clinical outcomes were measured with VAS score and ODI score. There was no difference between the two groups at baseline in VAS score and ODI score. The mean VAS scores at the 3-day was PPSR, 3.88 ± 1.07; OPSF, 6.89 ± 1.13 (*P* < 0.001), 3-month was PPSR, 2.33 ± 0.78; OPSF, 4.88 ± 1.37 (*P* < 0.001), and 12-month was PPSR, 0.92 ± 0.82; OPSF, 3.36 ± 1.45 (*P* < 0.001). The VAS and ODI scores in the PPSR group were notably lower than that in the OPSF group on follow-up (Figure 5A). This difference in scores indicate that PPSR was more effective than OPSF in improving fracture-induced short-term pain and long-term pain. In addition, the ODI score results demonstrated that ODI scores were lower in the PPSR group than in the OPSF group at the time point of 3-day (PPSR, 0.36 ± 0.11; OPSF, 0.59 ± 0.11, *P* < 0.001), 3-month (PPSR, 0.29 ± 0.11; OPSF, 0.44 ± 0.08, *P* < 0.001), and 12-month (PPSR, 0.22 ± 0.11; OPSF, 0.31 ± 0.08, *P* < 0.001) after surgery (Figure 5B). The results demonstrated that the quality of life of patients in the PPSR group was better than that of patients in the OPSF group.

## Discussion

Spine fracture injuries are quite common, with most occuring at thoracolumbar junction. According to an international statistic report, approximately 5% of spine fractures, and 54.9% thoracolumbar fractures inflict substantial financial burden (1, 10). Generally, treating most thoracolumbar fractures with neurologic deficits surgically is accepted widely (11). If no neurological dysfunction or instability of thoracolumbar fracture is observed in patients with thoracolumbar fractures, surgical intervention is not recommended. However, restoring the vertebral height and correcting the spinal kyphosis is indicated when the AVH loss exceeds 50% or when the local Cobb angle is greater than 15°–20° (12). OPSF is performed through stripping of paraspinal muscles, bilateral erector spine and multifidus muscles to expose vertebral plates, zygapophyses and transverse processes in thoracolumbar fracture. Although clinical symptoms can be significantly improved, a series of events such as intractable pain, stiffness, and weakness occur after the OPSF operation because of the denervation of the muscles, extensive adhesion and scar formation (9). Furthermore, OPSF plays a role in restoring the AVH and correcting the Cobb angle which is performed *via* longitude traction of the titanium rod. However, OPSF cannot effectively reduce the vertebrae fracture. These disadvantages have limited the wide use of OPSF in thoracolumbar fractures.

Over the last decades, minimally invasive spinal surgery has received increased attention. In 1984, Magerl firstly described the concept of percutaneous pedicle screw fixation (4). Percutaneous transpedicular screw fixation has fewer side effects on paraspinal muscles and can results in faster recovery than open fixation. After several improvements, Assaker reported that capability of percutaneous transpedicular fixation for treating thoracolumbar fractures (13). This technique became popular in treating thoracolumbar fractures because of the unique advantages such as shorter operative time, less blood loss, minor wound, and mild pains. It was reported that all 36 patients with thoracolumbar fractures who underwent minimally invasive percutaneous transpedicular fixation achieved satisfactory outcomes (14). However, the conventional percutaneous transpedicular technique uses Sextant's percutaneous fixation system that is less effective in reducing fractured vertebra than the open reduction internal fixation system (15). Generally, minimally invasive surgery is not recommended for patients with thoracolumbar fractures, who had greater than 50% vertebral height reduction since kyphosis cannot be adequately reduced (16). For thoracolumbar fractures without neurological deficit, decompression is not needed and the surgical intervention is focused on the restoration of the injured vertebra height and the correction of the spinal kyphosis caused by the fractured vertebra (9). For patients with thoracolumbar fractures, the decreased height of the vertebra can lead to changes in the sagittal spinal alignment and the spinal biomechanics. The increase in the kyphotic angle is a contributor to the instability of the fractured spinal segment and aggravates the deformity (17). Restoring the vertebral height and correcting the spinal kyphosis make sense when the AVH loss exceeds 50% or the local Cobb angle is greater than 15°–20° (12). However, the use of conventional percutaneous transpedicular fixation system cannot achieve these goals completely.

To address these concerns, PPSR, a novel minimally invasive internal fixation system was developed based on the conventional percutaneous transpedicular screw fixation system. PPSR was developed to restore and maintain the height of the injured vertebra and correct the spinal kyphosis resulting from vertebra fracture. PPSR can reconstruct the anterior column of the fractured vertebra by distracting the vertebra and transplant bone into the fractured vertebra. There are apparent advantages of using PPSR for treating thoracolumbar fractures without neurologic deficits. Firstly, the peroperative preoperative data in this study indicated that the operation duration, blood loss, postoperative stay, and the total costs of hospitalization in the PPSR group were notably lower than that in the OPSF group (18). The rate of complications between the two groups was similar, indicating the PPSR procedure had a safety profile that was manageable. Our results are similar with those of previous studies that have revealed that minimally invasive surgery result in various advantages such as shorter operation time, less blood loss, reduced hospital stay, decreased infection rate, and faster motor recovery (16). Secondly, the recovery rates of AVH of patients who underwent PPSR were significantly higher than that of patients in the OPSF group. The Cobb angle and the VWA results revealed that PPSR reduced kyphosis caused by the fractured vertebra. Both the recovery of the AVH and the correction of the Cobb angle and VWA in patients who underwent PPSR benefited from not only the titanium rods and screws (indirect longitudinal distraction) but also the bone transplantation in the fractured vertebra (direct distraction). In this present study, PPSR was more efficient in restoring AVH and improving the fracture-induced by spinal kyphosis than OPSF. This may be because OPSF was designed to achieve vertebral reduction only through the indirect longitudinal distraction effect of the titanium rods and screws. PPSR can restore spinal stability through restoring vertebral height and enhancing the biomechanical strength of the fractured vertebra. Early loss of correction following short-segment pedicle screw fixation (19) has been reported in a previous study. This finding differs with our study, where early loss of correction after PPSR or OPSF was not observed. Although clinical outcome observed during the follow-up period was satisfactory, longer duration of follow-up would also be beneficial.

Besides PPSR, there is a traditional kyphoplasty with percutaneous screw fixation that has long been used in treating thoracolumbar fractures (20). Although PPSR and the traditional kyphoplasty with percutaneous screw fixation utilize similar processes, PPSR has huge advantages. Firstly, bone graft materials that were used in PPSR have great osteoinductive potential that contribute to the bone healing of fractured vertebra, but the bone cement used in the kyphoplasty with percutaneous screw fixation cannot promote healing. Secondly, the incidence of the degeneration of the intervertebral discs in the adjacent segments in patients who accepted bone cement augmentation was high (21), due to weaker buffering role of bone cement than bone graft materials. Thirdly, the distraction of the fractured vertebra in the PPSR is slow and even cause in stable and reliable distraction effect. On the other hand, this distraction effect of the traditional kyphoplasty with percutaneous screw fixation is transient and elastic and it cannot achieve satisfactory distraction effect. Lastly, PPSR can significantly reduce medical costs because PPSR devices are not one-time medical consumable materials. Therefore, we believe that PPSR could provide great benefits to households and the society.

Initially, fixing of the spinal column involved the whole spine. With the development of concepts and technologies has led to focusing of fixation on the adjacent vertebrae of the involved segments rather than the whole spinal column. Then, to achieve a more precise therapeutic effect, Denis proposed the concept that the spinal column could be divided into three parts: anterior column, middle column, and posterior column (22). The development of this concept was from overall spinal column to the local segment. Consistently, based on the previous research findings, the newly-developed PPSR technique focuses on reconstructing the anterior column that comprises a key part of the spine. Data from this study has efficiently demonstrated the safety and efficacy of PPSR. PPSR is more effective than OPSF in improving the clinical outcomes of patients with thoracolumbar fractures by restoring vertebral height and correcting kyphosis more reliably.

Nevertheless, there are also some limitations in this study. Cases included in this study were patients with single-segment fracture, and therefore at present the role of PPSR in treating more complicated thoracolumbar fractures has not been elucidated. In addition, the evidence level of this retrospective study was quite low. A prospective, multicenter randomized clinical trial should be carried out. Moreover, other variable that may influence the therapeutic effect of PPSR, such as the causes of fracture and other types of fracture were not considered in the study. Also, the one-year follow-up period of this study was relatively brief. A longer follow-up period would have helped our team to obtain more precise results.

## Conclusions

In conclusion, the novel PPSR has a safety profile similar to OPSF. It is worth noting that PPSR restores the vertebral height and the spinal kyphotic angle better than the conventional open internal fixation. Thus, PPSR is a reliable option for treating thoracolumbar fractures.

## Data Availability

The raw data supporting the conclusions of this article will be made available by the authors, without undue reservation.

## References

[1] Hu R Mustard CA Burns C. Epidemiology of incident spinal fracture in a complete population. Spine. (1996) 21:492–9. 10.1097/00007632-199602150-00016 8658254

[2] Peev N Zileli M Sharif S Arif S Brady Z. Indications for nonsurgical treatment of thoracolumbar spine fractures: WFNS spine committee recommendations. Neurospine. (2021) 18:713–24. 10.14245/ns.2142390.195 35000324 PMC8752701

[3] Foley KT Gupta SK Justis JR Sherman MC. Percutaneous pedicle screw fixation of the lumbar spine. Neurosurg Focus. (2001) 10:E10. 10.3171/foc.2001.10.4.11 16732626

[4] Magerl FP. Stabilization of the lower thoracic and lumbar spine with external skeletal fixation. Clin Orthop Relat Res. (1984) 189:125–41. Available at: https://pubmed.ncbi.nlm.nih.gov/6478690/6478690

[5] Kocis J Kelbl M Kocis T Návrat T. Percutaneous versus open pedicle screw fixation for treatment of type A thoracolumbar fractures. Eur J Trauma Emerg Surg. (2020) 46:147–52. 10.1007/s00068-018-0998-4 30167741

[6] Li K Li Z Ren X Xu H Zhang W Luo D Effect of the percutaneous pedicle screw fixation at the fractured vertebra on the treatment of thoracolumbar fractures. Int Orthop. (2016) 40:1103–10. 10.1007/s00264-016-3156-9 26983411

[7] Siebenga J Leferink VJ Segers MJ Elzinga MJ Bakker FC Haarman HJ Treatment of traumatic thoracolumbar spine fractures: a multicenter prospective randomized study of operative versus nonsurgical treatment. Spine. (2006) 31:2881–90. 10.1097/01.brs.0000247804.91869.1e 17139218

[8] Burns JE Yao J Muñoz H Summers RM. Automated detection, localization, and classification of traumatic vertebral body fractures in the thoracic and lumbar spine at CT. Radiology. (2016) 278:64–73. 10.1148/radiol.2015142346 26172532 PMC4699497

[9] Yang M Zhao Q Hao D Chang Z Liu S Yin X. Comparison of clinical results between novel percutaneous pedicle screw and traditional open pedicle screw fixation for thoracolumbar fractures without neurological deficit. Int Orthop. (2019) 43:1749–54. 10.1007/s00264-018-4012-x 29909584

[10] Liu H Chen W Zhang J Jiang X Yang H Qu R Effects of pedicle screw number and insertion depth on radiographic and functional outcomes in lumbar vertebral fracture. J Orthop Surg Res. (2020) 15:572. 10.1186/s13018-020-02111-9 33256776 PMC7706188

[11] Singh R Rohilla RK Kamboj K Magu NK Kaur K. Outcome of pedicle screw fixation and monosegmental fusion in patients with fresh thoracolumbar fractures. Asian Spine J. (2014) 8:298–308. 10.4184/asj.2014.8.3.298 24967043 PMC4068849

[12] Jiang WQ Ke ZY Wu K Chen XL Lou ZQ. Effect of RTS versus percutaneous conventional pedicle screw fixation on type A thoracolumbar fractures: a retrospective cohort study. Eur Spine J. (2020) 29:2484–90. 10.1007/s00586-020-06418-3 32347391

[13] Assaker R. Minimal access spinal technologies: state-of-the-art, indications, and techniques. Joint Bone Spine. (2004) 71:459–69. 10.1016/j.jbspin.2004.08.006 15589424

[14] Ni WF Huang YX Chi YL Xu HZ Lin Y Wang XY Percutaneous pedicle screw fixation for neurologic intact thoracolumbar burst fractures. J Spinal Disord Tech. (2010) 23:530–7. 10.1097/BSD.0b013e3181c72d4c 21131801

[15] Wang HW Li CQ Zhou Y Zhang ZF Wang J Chu TW. Percutaneous pedicle screw fixation through the pedicle of fractured vertebra in the treatment of type A thoracolumbar fractures using sextant system: an analysis of 38 cases. Chin J Traumatol. (2010) 13:137–45. Available at: https://pubmed.ncbi.nlm.nih.gov/20515590/20515590

[16] Zhao Q Hao D Wang B. A novel, percutaneous, self-expanding, forceful reduction screw system for the treatment of thoracolumbar fracture with severe vertebral height loss. J Orthop Surg Res. (2018) 13:174. 10.1186/s13018-018-0880-4 29996932 PMC6042226

[17] Sadiqi S Verlaan JJ Lehr AM Chapman JR Dvorak MF Kandziora F Measurement of kyphosis and vertebral body height loss in traumatic spine fractures: an international study. Eur Spine J. (2017) 26:1483–91. 10.1007/s00586-016-4716-9 27497753

[18] Phan K Rao PJ Mobbs RJ. Percutaneous versus open pedicle screw fixation for treatment of thoracolumbar fractures: systematic review and meta-analysis of comparative studies. Clin Neurol Neurosurg. (2015) 135:85–92. 10.1016/j.clineuro.2015.05.016 26051881

[19] Perna A Santagada DA Bocchi MB Zirio G Proietti L Tamburrelli FC Early loss of angular kyphosis correction in patients with thoracolumbar vertebral burst (A3–A4) fractures who underwent percutaneous pedicle screws fixation. J Orthop. (2021) 24:77–81. 10.1016/j.jor.2021.02.029 33679031 PMC7910402

[20] Fuentes S Blondel B Metellus P Gaudart J Adetchessi T Dufour H. Percutaneous kyphoplasty and pedicle screw fixation for the management of thoraco-lumbar burst fractures. Eur Spine J. (2010) 19:1281–7. 10.1007/s00586-010-1444-4 20496038 PMC2989205

[21] Lu X Yang J Zhu Z Lv X Wu J Huang J Changes of the adjacent discs and vertebrae in patients with osteoporotic vertebral compression fractures treated with or without bone cement augmentation. Spine J. (2020) 20:1048–55. 10.1016/j.spinee.2020.02.012 32105771

[22] Denis F. Spinal instability as defined by the three-column spine concept in acute spinal trauma. Clin Orthop Relat Res. (1984) 189:65–76. Available at: https://pubmed.ncbi.nlm.nih.gov/6478705/6478705

